# Supplementary material for: Hydrogen sulfide as a potent predator-derived kairomone mediating fear-related responses in mice
Source: Commun Biol. 2025 Aug 1;8:1144. doi: 10.1038/s42003-025-08592-w (PMC12317040; doi:10.1038/s42003-025-08592-w)
Supplement: Supplementary file 2 — Supplementary information [file 42003_2025_8592_MOESM2_ESM.pdf]

Supplementary information

Hydrogen sulfide as a potent predator-derived kairomone mediating fear-related responses in mice

Ana Catarina Lopes, Julien Brechbühl, Aurélie de Vallière, Noah Gilliand, Flavio Ferreira and Marie-Christine Broillet

* Correspondence

to Marie-Christine Broillet, Email: marie-christine.broillet@unil.ch

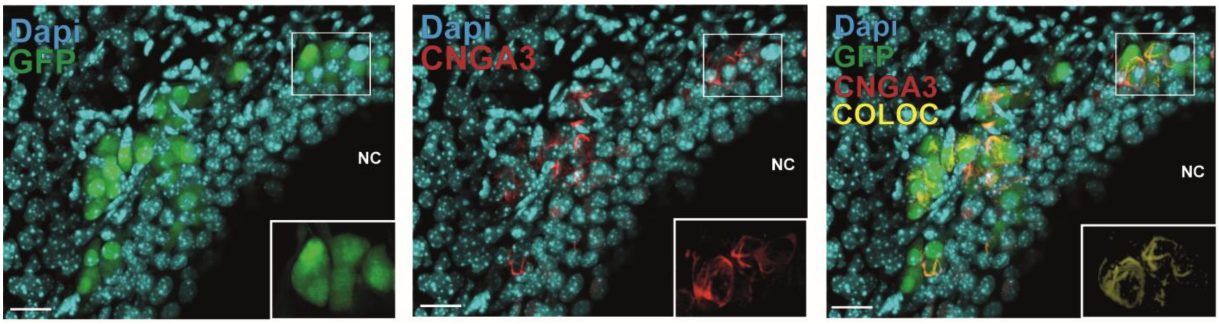

Supplementary Fig. 1 | CNGA3 channels are located in the cilia of the mouse GG neurons. Immunostainings performed on GG tissue slices (80 µm) from OMP-GFP mice. NC: nasal cavity. White squares: zoomed areas. Green, GFP expression in mature olfactory neurons. Red, CNGA3. Yellow, colocalization between GFP and CNGA3 expression (COLOC). Blue, Dapi staining. Scale bars: 20 µm.

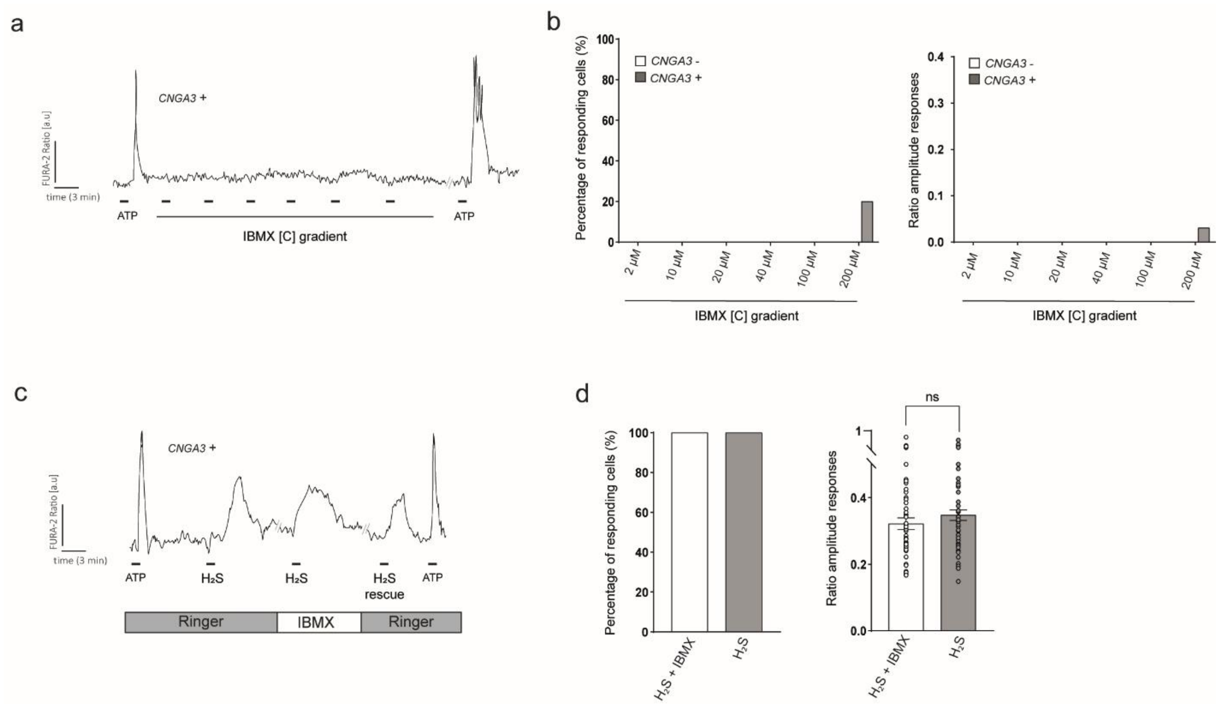

Supplementary Fig. 2 | Inhibition of PDE is not implicated in the H_2S responses observed in CNGA3 transfected HEK cells. a Representative example of intracellular calcium changes observed in one CNGA3-positive cell (CNGA3 +) in the presence of ATP (100 µM; to control the viability of the cells) and in the presence of different concentrations of IBMX (gradient). The Fura-2AM ratio is given in arbitrary units (a.u) and time in minutes. Bars indicate perfusion times. **b** Graphs representing the percentage of responding cells (left) and the amplitude of the responses (right) to different IBMX concentrations (from 2 to 200 µM). White bars, CNGA3-negative cells (CNGA3 -; Number of cells: 66); grey bars, CNGA3-positive cells (CNGA3 +; Number of cells: 40). **c** Intracellular calcium changes observed in one CNGA3-positive cell (CNGA3 +) in the presence of ATP (100 µM; to control the viability of the cells) and H_2S (25 µM) in a Ringer solution *vs*. in a Ringer solution containing IBMX (100 µM). The Fura-2AM ratio is given in arbitrary units (a.u) and time in minutes. Bars indicate perfusion times. **d** Graphs representing the percentage of responding cells (left) and the amplitude of the responses (right) in the presence of H_2S + IBMX (white bars) and H_2S (grey bars). Number of cells: 53. Data are represented as mean ± SEM with aligned dot plots and statistical analysis performed with an unpaired two-tailed Mann-Whitney test, ns for non-significant.

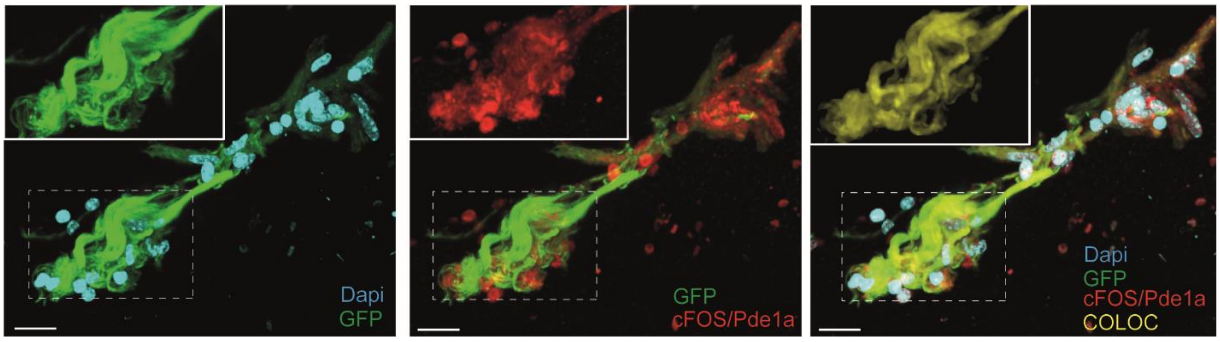

Supplementary Fig. 3 | Identification of the necklace glomeruli via their expression of Pde1a. Secondary immunostaining on tissue slices (100 µm) from the OMP-GFP mouse olfactory bulb. White dashed squares: zoomed areas. Necklace glomeruli (GFP in green) show expression of the Pde1a (anti-Pde1a in red). Blue, Dapi staining. Nuclei stained in blue are red due to residual cFOS staining from the first immunostaining. Colocalization of necklace glomeruli and Pde1a is shown in yellow (COLOC). Scale bars: 20 µm.

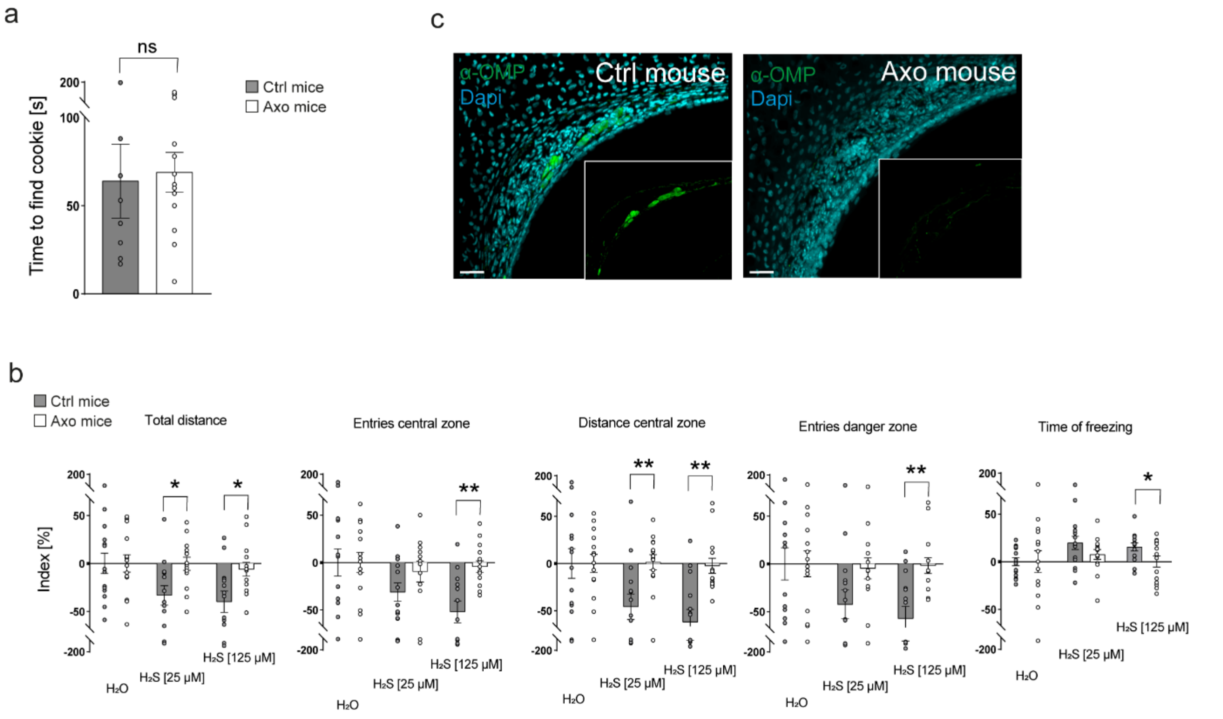

Supplementary Fig. 4 | Axo mice have no deficit in food detection, but they display a significant decrease in their fear-related behaviors. a No significant difference between Ctrl mice (n=8; grey bar) and Axo mice (n=14; white bar) was observed in the time to find an Oreo® cookie buried in the clean bedding of the cage. Data are represented as mean ± SEM with aligned dot plots and statistical analysis performed with unpaired Student's *t*-test was used for the analyses; ns: not significant (p=0.81). **b** Quantification of the stress-related behaviors observed in the presence of H_2O and H_2S (25 and 125 μM) displayed as indexes (%) for each parameter individually: the total distance travelled, the number of entries in the central zone, the distance travelled in the central zone, the number of entries in the danger zone and the total time freezing were quantified. n=14 mice for each phenotype (Ctrl mice (grey bars) and Axo mice (white bars)). Values are expressed as mean ± SEM; unpaired Student's *t*-test or Mann-Whitney test were used, * $p < 0.05$; ** $p < 0.01$. **c** Representative immunostaining on mouse GG tissue slices using α-OMP antibody (OMP, green) on C57BL/6 mice. On the right, axotomized (Axo) C57BL/6 mice showed no mature GG neurons. Nuclei are counterstained with Dapi (in blue). Scale bars: 50 μm.
